# Injectable Thermosensitive Composite Hydrogels for Sustained Nanoparticle Delivery and Enhanced Wound Healing

**DOI:** 10.3390/gels12030191

**Published:** 2026-02-25

**Authors:** Yiting Qiu, Zhiyun Cheng, Meiyan Liu, Dagui Zhang, Xia Gao, Longxiang Feng, Xianxiang Xu, Haoyang You, Xunxun Wu, Yong Diao

**Affiliations:** 1 College of Materials Science and Engineering, Huaqiao University, Xiamen 361021, China; qytdezh@163.com (Y.Q.); 1526211031@stu.hqu.edu.cn (D.Z.); 2 School of Medicine, Huaqiao University, Quanzhou 362021, China; chengzhiyun@hqu.edu.cn (Z.C.); 15863607363@163.com (M.L.); lxfengvon@gmail.com (L.F.); xuxianxiang@hqu.edu.cn (X.X.); youhaoyang@163.com (H.Y.); wuxunxun2015@163.com (X.W.); 3 College of Food Science and Engineering, Hainan University, Haikou 570100, China; gaoxia20202021@163.com

**Keywords:** injectable hydrogel, thermosensitive hydrogel, Pluronic F127, wound healing

## Abstract

Wound healing is frequently compromised by excessive oxidative stress, prolonged inflammation, and inadequate tissue regenerative capacity. To address these challenges, a thermosensitive and injectable composite hydrogel based on Pluronic F127 (F127), phosphatidylcholine (PC), and L-lysine (Lys) was developed for the sustained delivery of sinomenine–gallic acid nanoparticles (SGNPs) and the acceleration of wound repair. The hydrogel undergoes a rapid sol–gel transition at physiological temperatures through physical interactions, enabling excellent injectability and *in situ* gelation. The optimized composite hydrogel exhibited improved mechanical properties, enhanced structural stability, and a uniform porous microarchitecture. The F127−Lys−PCF127−Lys−PC@SGNPs hydrogel showed superior overall stability and hemocompatibility while enabling the sustained release of SGNPs for up to 24 h. Benefiting from the incorporation of SGNPs, the composite hydrogel displayed enhanced antioxidant activity, effectively scavenging free radicals and alleviating cellular oxidative stress. *In vitro* experiments demonstrated that the hydrogel promoted keratinocyte migration and proliferation. Furthermore, in a murine full-thickness skin wound model, treatment with F127−Lys−PCF127−Lys−PC@SGNPs significantly accelerated wound closure and facilitated re-epithelialization, angiogenesis, and collagen deposition. Collectively, this multifunctional thermosensitive hydrogel provides a promising platform for advanced wound dressings that integrate sustained delivery, antioxidant protection, and tissue regeneration.

## 1. Introduction

Mechanical injury is one of the most common types of trauma encountered in daily life, typically resulting from external forces such as blunt impact, friction, or accidental incisions. The extent of injury can range from superficial skin abrasions to severe damage involving deeper tissues [1]. As a critical barrier organ, the skin plays an indispensable role in preventing infection and maintaining physiological homeostasis. Once injured, the skin undergoes a highly coordinated wound healing process—typically comprising inflammatory, proliferative, and remodeling phases—to restore tissue structure and function [2]. Currently, wound management strategies are continuously evolving and include conventional clinical approaches such as growth factor delivery [3], gene therapy [4], stem cell-based therapies [5], and skin grafting [6]. Meanwhile, emerging technologies—including nanotherapeutics [7], hydrogels [8] and three-dimensional (3D) bioprinting [9]—have demonstrated considerable potential in enhancing tissue regeneration and improving healing outcomes. Among these advanced strategies, injectable hydrogels have attracted particular attention owing to their ability to mimic the extracellular matrix and conform more effectively to irregular wound geometries than traditional bulk hydrogels [10].

Pluronic F127 (also known as Poloxamer 407), a triblock copolymer (PEO-PPO-PEO) approved by the U.S. Food and Drug Administration (FDA) for pharmaceutical and biomedical applications, undergoes temperature-induced micellization and forms spherical microgels at approximately 37 °C [11]. However, F127-based hydrogels generally suffer from insufficient mechanical strength and limited adhesive properties [12,13]. To address these limitations, various modification strategies have been explored, including chemical crosslinking, the incorporation of physically reinforcing components, and the construction of functionalized network architectures. Beyond simple reinforcement, the construction of functionalized network architectures has emerged as a key strategy. In addition, the introduction of reversible crosslinked networks containing thermo-responsive, photo-responsive, pH-responsive or shear-responsive units can impart “smart” behaviors to hydrogels, enabling them to adapt to environmental changes and thereby improving performance under complex physiological conditions [14]. Compared with chemical crosslinking approaches, physical crosslinking strategies typically avoid the use of toxic crosslinkers, thereby reducing reagent residues and potential cytotoxicity [15]. L-lysine (Lys), an essential amino acid, not only promotes collagen synthesis and wound healing but also exhibits anti-inflammatory and antioxidant activities [16]. Lys has also been reported to improve drug solubility and transdermal delivery efficiency, while Lys-containing systems may facilitate nanoparticle transport across skin barriers through a “drag effect”, thereby enhancing local drug delivery [17]. Phosphatidylcholine (PC), a representative amphiphilic phospholipid, can interact with the PPO and PEO segments of F127 via hydrophobic interactions and hydrogen bonding [18].

Traditional Chinese medicines are widely available and cost-effective, making them an important source of bioactive therapeutic compounds. Sinomenine, an alkaloid extracted from the roots and stems of Sinomenium acutum, exhibits notable anti-inflammatory activity and has been reported to promote cell proliferation and migration, thereby accelerating wound healing [19]. Furthermore, Selvaraj et al. demonstrated that the co-loading of sinomenine hydrochloride and kaempferol hydrate into silk fibroin nanofibers not only endowed the system with strong antioxidant capacity but also significantly enhanced wound repair efficacy [20]. Gallic acid is a polyphenolic compound widely distributed in natural plants and is well known for its excellent antioxidant activity. It has been extensively investigated for applications in wound healing [21]. Sinomenine and gallic acid were assembled into sinomenine–gallic acid nanoparticles (SGNPs), which effectively improved the aqueous solubility of sinomenine and provided a multifunctional therapeutic platform with enhanced antioxidant and anti-inflammatory potential. The preparation and assembly process of SGNPs is schematically illustrated in Supplementary Figure S1.

In this study, Lys and PC were synergistically incorporated to construct a physically crosslinked, injectable F127−Lys−PCF127−Lys−PC composite hydrogel for the delivery of SGNPs, aiming to address the limited drug delivery efficiency of conventional F127-based hydrogels and to overcome the insufficient barrier function of traditional solid wound dressings. The physicochemical properties of the composite hydrogel were systematically characterized using Fourier transform infrared spectroscopy (FTIR), rheological analysis, thermogravimetric analysis (TGA), scanning electron microscopy (SEM), zeta potential measurement, and dynamic light scattering (DLS). *In vitro* wound healing-related biological performance was evaluated using HaCaT keratinocytes, while the *in vivo* wound healing efficacy was further assessed in a murine acute wound model.

## 2. Results and Discussion

### 2.1. Synthesis of Hydrogels

#### 2.1.1. Mechanical Analysis of Hydrogels

##### Formulation Optimization of the Composite Hydrogel

Based on the SGNPs prepared in this study (Supplementary Figure S1), a thermosensitive composite hydrogel was constructed by synergistically modulating F127 with PC and Lys to achieve *in situ* gelation and sustained delivery of SGNPs. According to previous reports, F127 exhibits a typical temperature-responsive sol–gel transition within a concentration range of 18–50% (*w*/*v*), while the incorporation of PC and Lys further enhances the mechanical stability of the hydrogel network. To elucidate the influence of auxiliary components on hydrogel stability, the effect of different PC contents on the system was first investigated. As shown in Supplementary Figure S2, excessive PC resulted in the formation of flocculent precipitates in the F127−Lys−PCF127−Lys−PC@SGNPs system. Therefore, the PC concentration was fixed at 0.1% (*w*/*v*). Based on this reported concentration range, various F127 concentrations were further evaluated experimentally (Table S1), and 25% (*w*/*v*) was selected based on gelation time, injectability and the requirement for rapid *in situ* gel formation in wound applications. Hydrogels with different Lys concentrations (0.3% and 0.6%, *w*/*v*) were prepared to obtain F127−Lys−PCF127−Lys−PC and F127−Lys−PCF127−Lys−PC@SGNPs formulations. The effects of Lys content on the viscoelastic properties of the hydrogels were subsequently investigated via rheological measurements (Supplementary Figure S3). The results indicate that all hydrogels exhibited typical viscoelastic characteristics, with the storage modulus (G′) exceeding the loss modulus (G″) within the low-strain region (<6%), suggesting gel-like behavior under ambient conditions [22]. Variations in G′ reflected the combined effects of Lys content and SGNP incorporation. At a Lys content of 0.3%, the introduction of SGNPs led to an increase in G′, whereas at a higher Lys concentration of 0.6%, SGNP loading resulted in a decrease in G′. Upon exceeding the critical strain, all hydrogels exhibited a rapid decline in both G′ and G″, indicating typical strain-softening behavior [23]. In the absence of SGNPs, G″ increased with increasing Lys content; however, after SGNP incorporation, the G″ value of the 0.6% Lys-containing F127−Lys−PCF127−Lys−PC@SGNPs hydrogel decreased, while the 0.3% Lys-containing F127−Lys−PCF127−Lys−PC@SGNPs formulation showed a relatively higher G″. The incorporation of SGNPs had no significant effect on tan δ. Based on these results, 0.3% Lys-containing F127−Lys−PCF127−Lys−PC@SGNPs was selected for subsequent studies. The concentration of SGNPs was defined based on the sinomenine content and fixed at 1 mM for all experiments, ensuring consistency across all subsequent characterizations. Consequently, the formulation comprising 25% (*w*/*v*) F127, 0.3% (*w*/*v*) Lys and 0.1% (*w*/*v*) PC was established as the optimized composition for the F127−Lys−PCF127−Lys−PC hydrogel system. Further hydrodynamic particle size and surface charge analyses showed that the diluted F127−Lys−PCF127−Lys−PC system exhibited an average hydrodynamic particle size of 273.40 ± 18.02 nm, a zeta potential of −50.58 ± 1.01 mV, and a PDI of 0.143 ± 0.02, indicating excellent dispersibility and colloidal stability of the system (Supplementary Figure S4).

##### Rheological Characterization of Hydrogels

**Figure 1:**
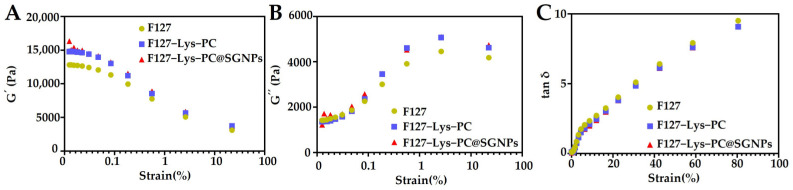
Rheological properties of F127-based hydrogels. (**A**) G′ as a function of strain. (**B**) G″ as a function of strain. (**C**) Strain-dependent loss factor (tan δ) of the hydrogels.

According to the formulation optimization in Section Formulation Optimization of the Composite Hydrogel, the 0.3% Lys and 0.1% PC concentrations were selected for further characterization. Subsequently, the rheological properties of pure F127, F127−Lys−PCF127−Lys−PC and F127−Lys−PCF127−Lys−PC@SGNPs (containing 1 mM sinomenine) were compared. The rheological properties of F127, F127−Lys−PCF127−Lys−PC and F127−Lys−PCF127−Lys−PC@SGNPs hydrogels were systematically evaluated. As shown in the rheological profiles (Figure 1A–C), all three hydrogels exhibited a G′ higher than the G″ at low strains (<8%), indicating that they behave as typical viscoelastic gels under ambient conditions. Compared with the pristine F127 hydrogel, the introduction of Lys and PC led to a marked increase in mechanical strength, with the initial G′ increasing from 12,816 Pa to 14,787 Pa, suggesting that these additives contribute to a stiffer hydrogel network. Upon the further incorporation of SGNPs, the G′ value of F127−Lys−PCF127−Lys−PC@SGNPs rose to 16,321 Pa, indicating that the presence of nanoparticles further reinforces the hydrogel matrix. In addition, when the applied strain exceeded 0.1%, the G″ values of both F127−Lys−PCF127−Lys−PC and F127−Lys−PCF127−Lys−PC@SGNPs surpassed that of the pure F127 hydrogel (Figure 1B). This suggests an enhanced energy dissipation capability in the composite hydrogels, which may be beneficial for maintaining structural integrity under external deformation. Meanwhile, compared with F127, the gelation point (G′ = G″) of the composite hydrogels shifted to higher values (Supplementary Figure S5), further indicating that the incorporation of Lys, PC and SGNPs contributes to the improved overall mechanical performance of the hydrogel system.

##### Thermosensitive Sol–Gel Transition Behavior

**Figure 2:**
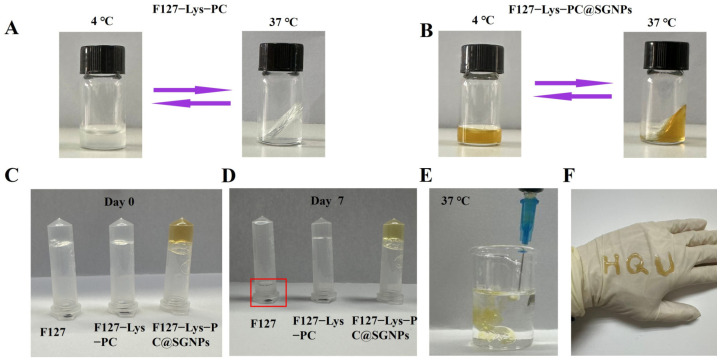
Thermoresponsive properties and injectability of F127-based hydrogels. (**A**) Reversible sol–gel transition of F127−Lys−PCF127−Lys−PC between 4 °C and 37 °C. (**B**) Reversible sol–gel transition of F127−Lys−PCF127−Lys−PC@SGNPs between 4 °C and 37 °C. (**C**) Macroscopic appearance of hydrogels on Day 0. (**D**) Hydrogel stability after 7 days of storage at 25 °C. The red square highlights the sedimentation of pure F127 due to its insufficient viscosity and inability to adhere to the tube wall. (**E**) *In situ* gel formation of the hydrogel upon injection into PBS at 37 °C. (**F**) Injectable shaping behavior of the hydrogel on a glove surface.

The as-prepared F127−Lys−PCF127−Lys−PC@SGNPs hydrogel precursor exhibited the appearance of a semi-transparent and homogeneous solution at 4 °C, facilitating handling and transfer. The preparation process was straightforward and demonstrated excellent reproducibility. As shown in Figure 2A,B, F127−Lys−PCF127−Lys−PC@SGNPs exhibited a pronounced sol–gel transition in response to temperature fluctuations. When the temperature increased from 4 °C to physiological temperature (37 °C), the liquid precursor gradually transformed into a stable semi-solid gel. This transition is primarily attributed to the thermally induced micellization and subsequent packing of F127 chains [24].

##### Injectability and Structural Stability of Hydrogels

As shown in Figure 2C,D, at the initial time point (day 0), all three hydrogels in their optimized formulations—F127, F127−Lys−PCF127−Lys−PC, and F127−Lys−PCF127−Lys−PC@SGNPs—successfully formed intact and stable gel structures at 37 °C. After storage at room temperature for 7 days, the pure F127 hydrogel exhibited structural collapse due to its relatively low viscosity, whereas both composite hydrogels maintained excellent self-supporting ability and structural integrity, without observable phase separation or syneresis. The incorporation of SGNPs did not disrupt the three-dimensional network of the F127−Lys−PCF127−Lys−PC hydrogel; furthermore, F127−Lys−PCF127−Lys−PC@SGNPs remained structurally stable at room temperature for at least one month, suggesting excellent long-term stability. In addition, the hydrogel precursor was smoothly extruded through a syringe into PBS or onto the dorsal skin of the hand (Figure 2E,F), demonstrating its superior injectability. Collectively, these results suggest that the F127−Lys−PCF127−Lys−PC@SGNPs hydrogel possesses both thermosensitive and injectable characteristics, enabling rapid gelation at physiological temperatures and effective adaptation to irregular wound geometries [25].

### 2.2. Structural Characterization and Analysis

#### 2.2.1. FTIR Spectral Analysis

**Figure 3:**
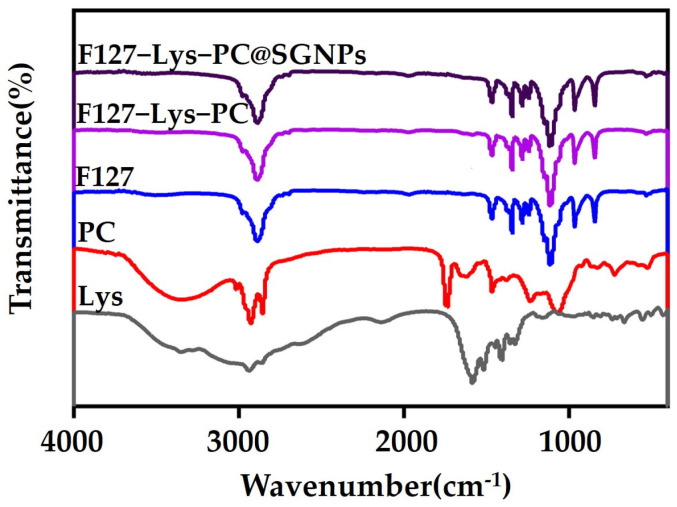
FTIR spectra of Lys, PC, F127, F127−Lys−PCF127−Lys−PC and F127−Lys−PCF127−Lys−PC@SGNPs.

To investigate the molecular interactions among different components in the F127−Lys−PCF127−Lys−PC@SGNPs system and verify the successful construction of the composite hydrogel, FTIR spectroscopy was employed (Figure 3). The FTIR spectrum of pristine F127 displayed characteristic absorption bands corresponding to the stretching vibration of -CH_3_ groups at 2888 cm^−1^ and the C-O-C stretching vibration of the PEO-PPO backbone at 1110 cm^−1^, confirming the typical structural features of F127. In comparison, no distinct characteristic peaks attributable to Lys, PC, or SGNPs were observed in the spectra of F127−Lys−PCF127−Lys−PC and F127−Lys−PCF127−Lys−PC@SGNPs, which is likely due to their relatively low concentrations and the dominance of the strong absorption signals from F127 [26]. Overall, the FTIR spectra of F127−Lys−PCF127−Lys−PC and F127−Lys−PCF127−Lys−PC@SGNPs showed no new characteristic absorption bands or significant peak shifts compared to those of pristine F127, indicating that the introduction of Lys, PC, and SGNPs did not induce noticeable changes in the chemical structure of the F127 backbone. These results suggest that the formation of the hydrogel network is primarily governed by non-covalent interactions, such as physical entanglement and intermolecular forces, rather than the generation of new chemical bonds [13].

#### 2.2.2. TGA

**Figure 4:**
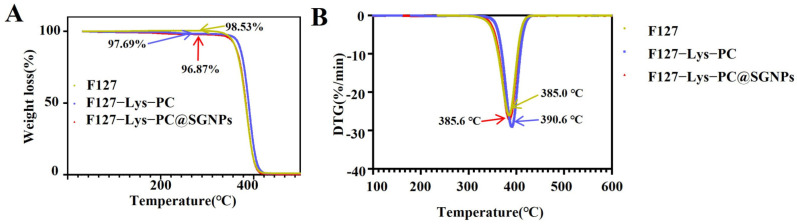
(**A**) TGA curves of F127, F127−Lys−PC and F127−Lys−PC@SGNPs. (**B**) Corresponding DTG curves illustrating the thermal decomposition behavior.

TGA was subsequently performed to evaluate the thermal stability of the three hydrogels. As shown in Figure 4A, the TGA curves of F127, F127−Lys−PCF127−Lys−PC and F127−Lys−PCF127−Lys−PC@SGNPs, together with their corresponding DTG profiles (Figure 4B), all exhibit characteristic single-step thermal degradation behavior, which is consistent with previously reported degradation patterns of F127-based materials [27]. At 330 °C, pristine F127 showed a weight loss of 1.47 ± 0.08%, whereas the initial weight losses of F127−Lys−PCF127−Lys−PC and F127−Lys−PCF127−Lys−PC@SGNPs were 2.31 ± 0.12% and 3.13 ± 0.15%, respectively, following a similar trend. During the later degradation stages, the incorporation of SGNPs may alter the packing arrangement of polymer chains and the intermolecular interactions within the original F127−Lys−PCF127−Lys−PC network, thereby partially reducing the thermal stabilization effect of the composite system. Given the absence of new characteristic peaks or peak shifts in the FTIR spectra, the variation in thermal stability is therefore attributed primarily to changes in physical chain organization rather than chemical bonding. DTG analysis revealed that the maximum decomposition peak of F127 was located at 385.0 °C. Upon the cooperative incorporation of Lys and PC, the DTG peak of F127−Lys−PCF127−Lys−PC shifted to a higher temperature (390.6 °C), indicating the enhanced thermal stability of the composite network. This improvement might be attributed to intermolecular interactions, such as hydrogen bonding or electrostatic interactions, between F127 chains and the amino or phospholipid moieties of Lys and PC. After the further loading of SGNPs, the DTG peak of F127−Lys−PC@SGNPs slightly decreased to 385.6 °C, approaching that of pristine F127. This result suggests that the introduction of SGNPs partially offsets the thermal stability enhancement conferred by Lys and PC, which is consistent with the observations from the TGA curves.

#### 2.2.3. SEM Analysis

**Figure 5:**
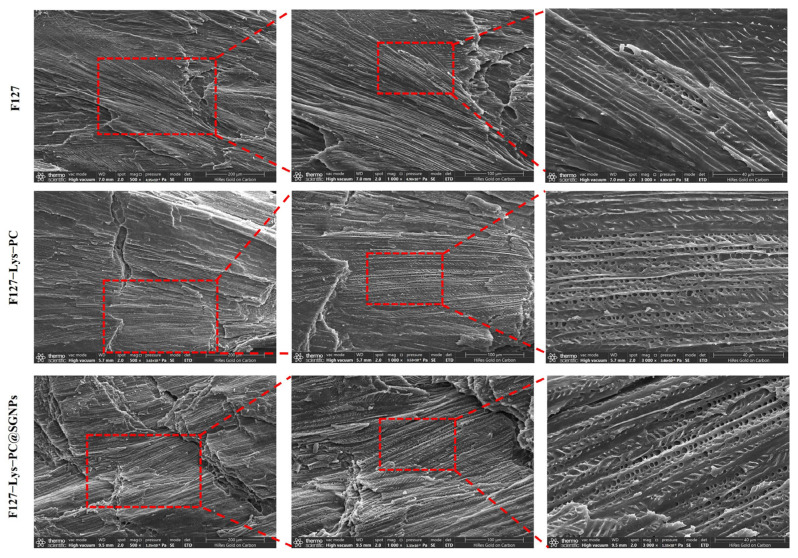
SEM images showing the cross-sectional morphologies of freeze-dried F127, F127−Lys−PC, and F127−Lys−PC@SGNPs hydrogels. Enlarged views of the regions marked by red dashed boxes reveal the internal porous architectures at different magnifications. The scale bars correspond to 200 μm, 100 μm, and 40 μm from left to right, respectively. All samples were prepared by liquid nitrogen fracturing followed by gold sputtering.

After freeze-drying, the hydrogels were fractured in liquid nitrogen to obtain cross-sectional samples, and their internal microstructures were examined via SEM (Figure 5). SEM observations of the freeze-dried pristine F127 hydrogel revealed a relatively irregular internal morphology, characterized by discontinuous and non-uniform pore distribution. This morphology can be primarily attributed to the intrinsic limitations of unmodified F127 in forming a stable three-dimensional crosslinked network. Instead, the gel structure of F127 is predominantly governed by thermoresponsive micellar aggregation and reversible physical entanglements between polymer chains; the lack of effective crosslinking support consequently leads to limited network stability and structural controllability [28]. In contrast, under the synergistic regulation of Lys and PC, the F127−Lys−PC hydrogel exhibited a more uniform and regular porous network morphology, with continuously distributed and well-interconnected pores. These observations suggest that the introduction of the composite components facilitates the formation of a more homogeneous and stable three-dimensional network within the F127-based hydrogel. The resulting porous architecture is favorable for mass transport and tissue infiltration, providing a structural basis for its application as a wound dressing [29]. After the further incorporation of SGNPs, the F127−Lys−PC@SGNPs hydrogel maintained an intact three-dimensional porous structure, with no obvious pore collapse or network disruption observed. This indicates that the loading of SGNPs does not adversely affect the network architecture of the composite hydrogel. Overall, these results demonstrate that the hydrogel system preserves excellent internal microstructural stability while enabling effective nanoparticle incorporation.

To quantify these observations, the porosity was measured using ImageJ version 1.54p (National Institutes of Health, Bethesda, MD, USA), running on Java 1.8.0_345 (64-bit). (Figure S6). The results showed that the porosity of pristine F127 was 11.55 ± 6.38%, which increased to 32.99 ± 6.19% for F127−Lys−PC and 30.14 ± 2.81% for F127−Lys−PC@SGNPs. These quantitative data are consistent with the SEM morphologies, confirming that the modification facilitates a more interconnected porous network.

#### 2.2.4. Determination of Particle Size, Polydispersity Index (PDI) and Zeta Potential

The average hydrodynamic size, PDI, and zeta potential of the SGNP-containing hydrogel system (F127−Lys−PC@SGNPs) were characterized by DLS. It should be noted that these measurements were performed on the hydrogel dispersion as a whole, reflecting the apparent nanoscale structural domains formed within the nanoparticle-loaded hydrogel system, rather than isolated SGNPs. This analysis was conducted to evaluate the colloidal stability and dispersion characteristics of the nanostructured hydrogel system, which are relevant to its storage stability and biomedical application.

**Figure 6:**
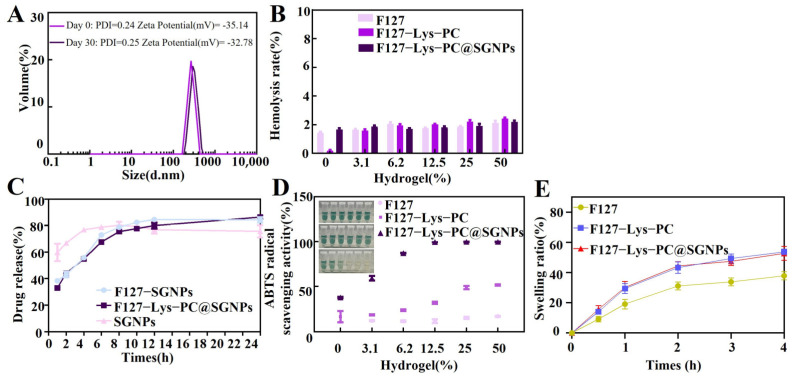
Stability, hemocompatibility, drug release, and antioxidant activity of the hydrogels. (**A**) Particle size distribution, zeta potential, and polydispersity index (PDI) of the hydrogel measured on Day 0 and after 30 days of storage at 4 °C. (**B**) Hemolysis ratios of F127, F127−Lys−PCF127−Lys−PC and F127−Lys−PCF127−Lys−PC@SGNPs hydrogels at different concentrations, indicating blood compatibility. (**C**) *In vitro* cumulative drug release profiles of the hydrogels under simulated physiological conditions. (**D**) Antioxidant activity of the hydrogels evaluated by the ABTS radical scavenging assay. (**E**) The swelling ratio.

As shown in Figure 6A, these parameters were measured for the hydrogel stored at 4 °C on Day 0 and Day 30, respectively. The results indicated that no significant changes in particle size distribution or surface charge occurred over the storage period, suggesting that the system exhibits good physical stability under low-temperature conditions. Zeta potential is a critical indicator of the surface charge characteristics of nanoparticles, their colloidal stability, and their potential interactions with biological systems. Generally, an absolute zeta potential value greater than 30 mV is considered sufficient to provide adequate repulsion between particles, thereby effectively preventing aggregation and maintaining dispersion stability [30]. The PDI is used to evaluate the uniformity of particle size distribution, with lower values indicating a more homogeneous and stable system [31]. The F127−Lys−PCF127−Lys−PC@SGNPs system exhibited an average hydrated particle size of 264.91 ± 2.50 nm, a PDI of 0.25 ± 0.016, and a zeta potential of −35.14 ± 2.95 mV. These results demonstrate that the nanoparticle-loaded hydrogel system possesses a relatively uniform particle size distribution and robust colloidal stability. The moderate particle size is favorable for diffusion and local retention within biological tissues, while the relatively high negative zeta potential contributes to enhanced dispersion stability during storage and application, which is crucial for achieving stable delivery and controlled release.

### 2.3. In Vitro Biocompatibility and Functional Evaluation

#### 2.3.1. Hemocompatibility Evaluation of Hydrogels

Since hydrogel dressings are intended for direct contact with wound tissues during practical application, biocompatibility is a critical prerequisite for evaluating their clinical potential [32]. In this study, the hemocompatibility of different hydrogel formulations was further assessed via hemolysis assays. As shown in Figure 6B, the hemolysis rates of F127, F127−Lys−PCF127−Lys−PC, and F127−Lys−PCF127−Lys−PC@SGNPs at various tested concentrations were all below the 5% threshold specified by the international standard ISO 10993-4 [33] indicating excellent hemocompatibility of these hydrogel materials [34]. These results suggest that the incorporation of Lys, PC and SGNPs does not cause a noticeable reduction in blood compatibility. The composite hydrogel system maintains good biosafety while achieving enhanced functionality, providing experimental support for its further application in wound repair and other biomedical fields.

#### 2.3.2. *In Vitro* Release

*In vitro* release studies were conducted to compare the release behaviors of free SGNPs and SGNPs encapsulated within different hydrogel matrices, with the aim of evaluating the regulatory effect of the hydrogel systems on drug release kinetics and sustained release performance [35]. The amount of released sinomenine was quantified by UV–vis spectrophotometry based on a calibration curve constructed using standard sinomenine solutions over an appropriate concentration range. The concentration of released sinomenine was quantified using a calibration curve constructed over a concentration range of 1–5 mg/mL, which exhibited good linearity (y = 7042x + 3444, R^2^ = 0.9981) (Supplementary Figure S7). As shown in Figure 6C, the *in vitro* release profiles of free SGNPs, F127-SGNPs and F127−Lys−PCF127−Lys−PC@SGNPs were systematically compared. The results indicated that free SGNPs, owing to their high water solubility, exhibited rapid release kinetics, reaching a release plateau within 8 h. In contrast, F127-SGNPs achieved prolonged release over approximately 12 h, which can be attributed to the encapsulation of SGNPs within the F127 matrix that restricted diffusion. Notably, F127−Lys−PCF127−Lys−PC@SGNPs displayed the most pronounced sustained release behavior, with the release duration extended to 24 h. This enhanced release control is primarily attributed to the effective entrapment of SGNPs within the F127−Lys−PCF127−Lys−PC composite network, which increases the diffusion path length and slows down drug transport. Consequently, the composite hydrogel system prolongs drug retention and potentially improves delivery efficiency [36].

#### 2.3.3. ABTS^+^ Free Radical Scavenging Activity

During the initial inflammatory phase of wound healing, reactive oxygen species (ROS) are produced to eliminate invading pathogens. However, excessive ROS generation beyond the intrinsic antioxidant capacity of tissues can lead to local oxidative stress, which may impair the wound healing process [37,38]. To quantitatively assess the antioxidant properties of the hydrogels developed in this study, the ABTS assay was employed to evaluate their free radical scavenging activity. Three hydrogel formulations—F127, F127−Lys−PCF127−Lys−PC (without SGNPs), and F127−Lys−PCF127−Lys−PC@SGNPs (containing 1 mM sinomenine at 100% hydrogel)—were evaluated, and each formulation was tested at a series of dilutions to assess concentration-dependent scavenging behavior. As shown in Figure 6D, pristine F127 exhibited limited antioxidant activity. Following the incorporation of Lys and PC, a moderate increase in antioxidant activity was observed for F127−Lys−PCF127−Lys−PC, which may be attributed to the inherent antioxidant properties of Lys and PC. Upon further loading of SGNPs, the composite hydrogel system exhibited a substantially enhanced radical scavenging capacity. At a concentration of 6.25%, the ABTS radical scavenging rate of F127 was 11.6 ± 0.98%, which increased to 23.67 ± 0.85% for F127−Lys−PCF127−Lys−PC, while F127−Lys−PCF127−Lys−PC@SGNPs reached 87.14 ± 0.29%. When the concentration was increased to 12.5%, the scavenging efficiency of F127−Lys−PCF127−Lys−PC@SGNPs approached near-total radical elimination (99.54 ± 0.08%). These results suggest that the incorporation of SGNPs markedly improves the antioxidant performance of the composite hydrogel system.

#### 2.3.4. Swelling Ratio

The water uptake behavior of F127, F127−Lys−PCF127−Lys−PC and F127−Lys−PCF127−Lys−PC@SGNPs hydrogels was evaluated to further assess their hydration characteristics and internal network structure. As shown in Figure 6E, all hydrogels exhibited a rapid increase in water uptake during the initial stage, followed by a gradual approach to equilibrium within 4 h. Compared with pristine F127, the incorporation of Lys and PC markedly enhanced the water uptake capacity of the hydrogel, indicating increased hydrophilicity and a more open network structure. The F127−Lys−PCF127−Lys−PC@SGNPs hydrogel showed a comparable or slightly higher water uptake than F127−Lys−PCF127−Lys−PC, suggesting that the introduction of SGNPs did not compromise the swelling behavior of the composite system. Overall, these results demonstrate that the composite hydrogels possess improved water absorption ability, which is favorable for maintaining a moist wound environment.

#### 2.3.5. Cell Migration and Antioxidant Activity

**Figure 7:**
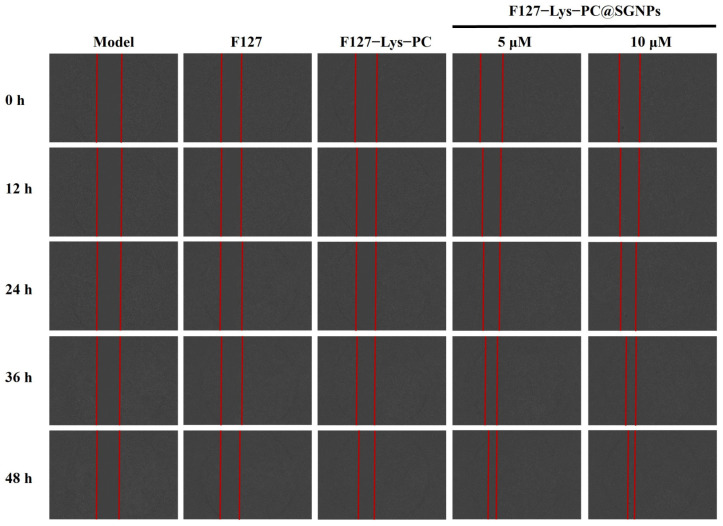
*In vitro* scratch wound healing assay of HaCaT cells. Representative images showing HaCaT cell migration at 0, 12, 24, 36 and 48 h after treatment with Model, F127, F127−Lys−PCF127−Lys−PC and F127−Lys−PCF127−Lys−PC@SGNPs (containing 5 and 10 μM sinomenine). The red lines indicate the initial scratch boundaries.

**Figure 8:**
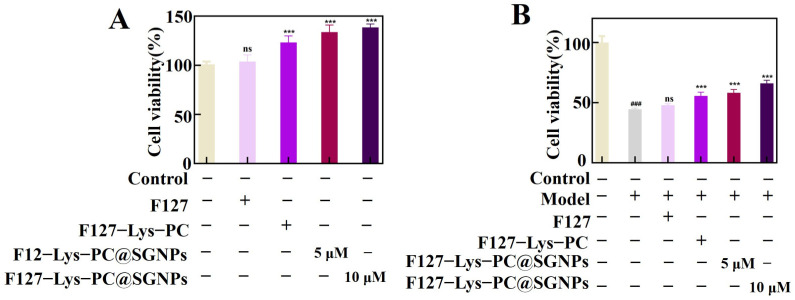
Effects of different hydrogels on HaCaT cell viability and protection against oxidative stress. (**A**) Cell viability of HaCaT cells after 24 h incubation with different hydrogel formulations, evaluated by the CCK-8 assay to assess cytocompatibility. *** *p* < 0.001 vs. control group and ns. statistically not significant. (**B**) Protective effects of the hydrogels on HaCaT cell viability in an H_2_O_2_-induced oxidative injury model, assessed by the CCK-8 assay. HaCaT cells were pretreated with different hydrogels prior to H_2_O_2_ exposure. ### *p* < 0.001 vs. control group; *** *p* < 0.001 vs. model group and ns. statistically not significant. Data are presented as mean ± SD (*n* = 6).

To investigate the effect of F127−Lys−PCF127−Lys−PC@SGNPs on wound healing-related cellular behavior, a scratch assay was performed using HaCaT keratinocytes. As shown in the results (Figure 7), compared with the model control group, the pristine F127 hydrogel exhibited negligible capacity to promote cell migration. In contrast, the F127−Lys−PCF127−Lys−PC composite hydrogel moderately enhanced cell migration, likely due to the synergistic effects of Lys and PC. Notably, the F127−Lys−PCF127−Lys−PC@SGNPs composite hydrogel displayed a markedly enhanced pro-migratory effect, as evidenced by a significantly accelerated scratch closure rate. These findings suggest that F127−Lys−PCF127−Lys−PC@SGNPs can effectively promote keratinocyte migration, a critical process in epidermal regeneration and wound repair. Subsequently, the effects of different hydrogel formulations on HaCaT cell proliferation within 24 h were evaluated using the CCK-8 assay (Figure 8A). The results showed that F127−Lys−PCF127−Lys−PC increased the cell proliferation rate to 123.18 ± 6.62%, likely benefiting from the combined effects of Lys and PC. In comparison, F127−Lys−PCF127−Lys−PC@SGNPs exhibited a more pronounced proliferative effect, with cell viabilities of 133.74 ± 7.18% at low dosages and 138.51 ± 3.44% at high dosages. This trend was consistent with the results of the scratch assay, further indicating that the SGNP-loaded composite hydrogel may play a beneficial role in promoting keratinocyte regeneration and wound healing [39].

**Figure 9:**
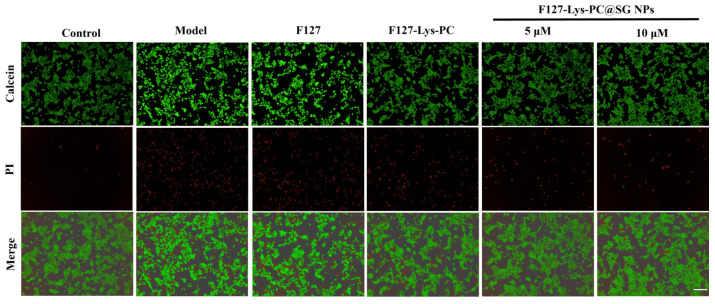
Protective effects of F127, F127–PC–Lys and F127−Lys−PCF127−Lys−PC@SGNPs hydrogels on H_2_O_2_-induced oxidative injury in HaCaT cells. Live/dead staining was performed using Calcein-AM (green, live cells) and propidium iodide (PI, red, dead cells). Merged images illustrate overall cell viability under different treatments. Scale bar: 200 μm.

**Figure 10:**
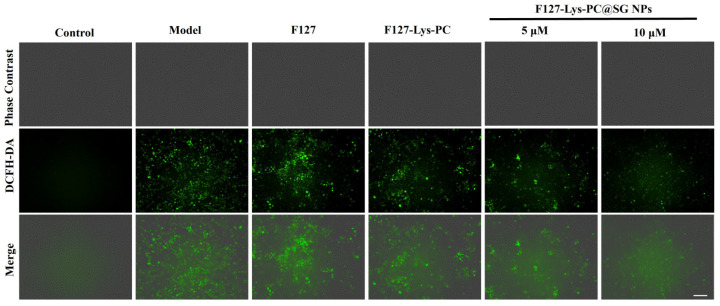
ROS levels in H_2_O_2_-injured HaCaT cells after treatment with F127, F127–PC–Lys and F127−Lys−PCF127−Lys−PC@SGNPs hydrogels, visualized by DCFH-DA staining and merged with bright-field images. Scale bar: 200 μm.

Furthermore, an H_2_O_2_-induced oxidative injury model was established at the cellular level using HaCaT cells to evaluate the antioxidant protective effects of the hydrogels. As indicated by cell viability assays (Figure 8B) and live/dead staining results (Figure 9), F127−Lys−PCF127−Lys−PC@SGNPs effectively attenuated H_2_O_2_-induced cell death and improved overall cell viability. In addition, intracellular ROS levels were assessed using the DCFH-DA fluorescent probe (Figure 10). The results demonstrated that treatment with F127−Lys−PCF127−Lys−PC@SGNPs markedly suppressed the excessive ROS generation induced by H_2_O_2_, thereby alleviating intracellular oxidative stress. Taken together, these findings suggest that the composite hydrogel exerts potent cytoprotective effects under oxidative stress conditions and may contribute to improved cellular responses essential for wound healing, highlighting its potential applicability in wound repair [40].

### 2.4. In Vivo Wound Healing Performance

#### Efficacy of Hydrogels in Wound Healing

**Figure 11:**
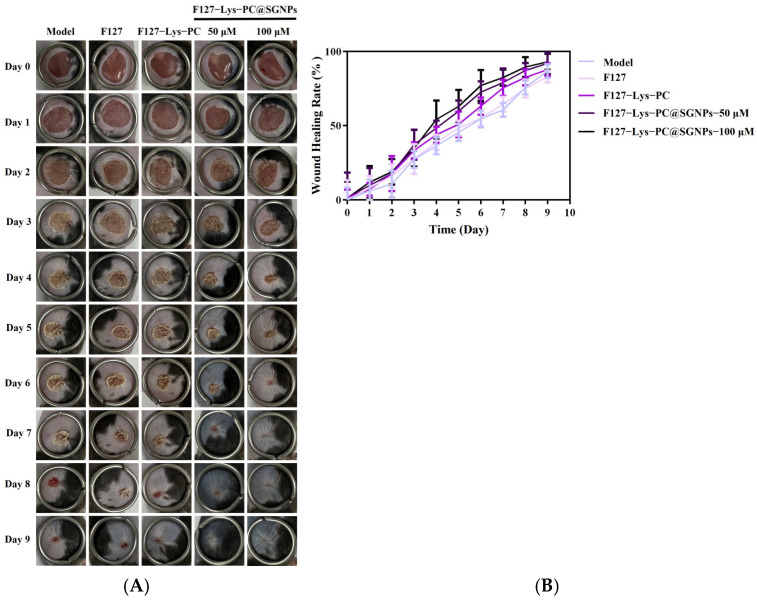
*In vivo* wound healing evaluation in a murine full-thickness skin wound model. (**A**) Representative photographs of wounds in mice treated with Model, F127, F127−Lys−PCF127−Lys−PC and F127−Lys−PCF127−Lys−PC@SGNPs from Day 0 to Day 9. (**B**) Quantitative analysis of wound healing rate over time for the different treatment groups.

To further evaluate the *in vivo* therapeutic efficacy of F127−Lys−PCF127−Lys−PC@SGNPs, a full-thickness skin wound model was established in mice. The experimental procedure was conducted according to the method described by Diao et al. [41]. Briefly, mice were anesthetized with isoflurane, and a circular full-thickness skin wound (approximately 1 cm in diameter) was created on the dorsal region using sterile surgical scissors. The wounds were subsequently treated daily with the designated formulations, and the wound healing process was monitored and recorded at predetermined time points. Untreated wounds served as the model control group, while additional treatment groups included F127, F127−Lys−PCF127−Lys−PC and F127−Lys−PCF127−Lys−PC@SGNPs containing SGNPs at two concentrations (50 μM and 100 μM of sinomenine). As shown in the results, compared with the F127 and F127−Lys−PCF127−Lys−PC groups, treatment with F127−Lys−PCF127−Lys−PC@SGNPs at both doses resulted in a significantly more rapid wound closure process, indicating superior wound healing performance (Figure 11).

**Figure 12:**
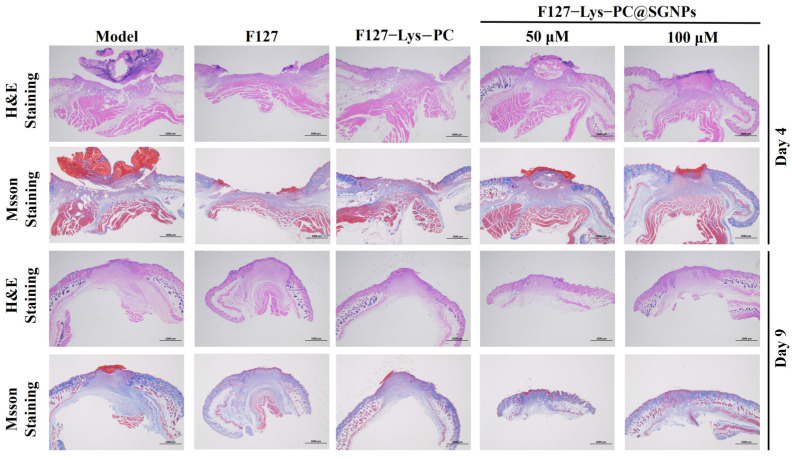
Histological evaluation of wound tissues in the murine wound healing model. Representative hematoxylin and eosin (H&E) and Masson’s trichrome-stained sections of wound tissues harvested on Day 4 and Day 9 after different treatments. Scale bar: 1000 μm.

Skin tissues were harvested on Days 4 and 9 post-treatment and subjected to hematoxylin and eosin (H&E) staining and Masson’s trichrome staining to evaluate tissue repair and regeneration (Figure 12). H&E staining results revealed that, compared with the F127 and F127−Lys−PCF127−Lys−PC groups, the F127−Lys−PCF127−Lys−PC@SGNPs-treated group exhibited a more developed epidermal structure as early as Day 4. In contrast, wounds treated with F127 and F127−Lys−PCF127−Lys−PC predominantly showed discontinuous or incomplete epidermal coverage at the same time point, indicating a delayed healing process. By Day 9, the F127−Lys−PCF127−Lys−PC@SGNPs group displayed significant maturation of the newly formed epidermis, accompanied by more prominent neovascularization within the dermal layer, whereas the control groups still exhibited inadequate epidermal regeneration and tissue reconstruction. Consistent with these observations, Masson’s trichrome staining demonstrated earlier and more pronounced collagen deposition in the F127−Lys−PCF127−Lys−PC@SGNPs group compared with the other groups. The intense blue staining indicated denser and more mature collagen fiber organization, suggesting enhanced collagen synthesis and ordered tissue remodeling. These histological findings suggest that the composite hydrogel promotes wound repair by providing a moist and protective microenvironment at the wound site while enabling the sustained release of Lys, PC and SGNPs. The combined effects of these components synergistically enhance keratinocyte migration and proliferation, thereby facilitating tissue regeneration and wound healing [42].

## 3. Conclusions

In conclusion, we successfully developed a physically crosslinked, thermosensitive injectable hydrogel based on F127, Lys and PC for the localized delivery of SGNPs in wound healing. Synergistic supramolecular assembly endowed the F127−Lys−PCF127−Lys−PC@SGNPs hydrogel with enhanced mechanical stability, excellent injectability, and sustained drug release under physiological conditions. Functionally, the hydrogel established a pro-regenerative wound microenvironment by providing prolonged antioxidant protection, promoting keratinocyte migration and proliferation, and accelerating wound closure, re-epithelialization, collagen deposition, and neovascularization in a murine full-thickness wound model. Overall, this work demonstrates that biocompatible physical interactions can effectively overcome the intrinsic limitations of conventional Pluronic hydrogels without the need for chemical crosslinking, offering a safe and versatile injectable platform for wound repair and broader tissue engineering applications.

## 4. Materials and Methods

### 4.1. Materials

F127 and Lys were purchased from Aladdin (Shanghai, China). PC (from soybean, >98%), potassium persulfate (99.5%), and 2,2′-azino-bis(3-ethylbenzothiazoline-6-sulfonic acid) diammonium salt (ABTS) were obtained from Macklin (Shanghai, China). SGNPs were prepared according to the method detailed in the Supplementary Information (Figure S1). HaCaT cells and HaCaT cell-specific culture media were purchased from Procell Life Science & Technology Co., Ltd. (Hubei, China). Trypsin-EDTA solution (0.25% trypsin), Cell Counting Kit-8 (CCK-8), the Calcein-AM/propidium iodide (PI) live/dead staining kit, and the ROS assay kit were purchased from Beyotime Biotechnology (Shanghai, China).

### 4.2. Preparation of Hydrogels

The F127−Lys−PCF127−Lys−PC hydrogel was prepared via a liquid–liquid dispersion method. Briefly, the PC was first dispersed in double-distilled water at 25 °C and magnetically stirred until a homogeneous dispersion was obtained. Subsequently, the Lys solution was added, and the mixture was continuously stirred to ensure uniform mixing. The resulting solution was then cooled to 4 °C under constant stirring, during which 25% (*w*/*v*) F127, pre-dissolved in double-distilled water at 4 °C, was slowly added. The mixture was stirred overnight at 4 °C until a transparent F127−Lys−PCF127−Lys−PC hydrogel was formed. The F127−Lys−PCF127−Lys−PC@SGNPs composite hydrogel was prepared following a similar liquid–liquid dispersion strategy. Briefly, the PC was added to an aqueous SGNPs solution and magnetically stirred at 25 °C for 2 h to allow for sufficient interaction between the PC and SGNPs. Subsequently, the Lys solution and F127 were sequentially added following the aforementioned procedure. The mixture was continuously stirred overnight at 4 °C, yielding a uniform F127−Lys−PCF127−Lys−PC@SGNPs composite hydrogel. The detailed chemical compositions and the final concentrations of all components in the hydrogel formulations are summarized in Table 1.

### 4.3. FTIR Spectroscopy

FTIR spectroscopy was employed to characterize the chemical structures of the freeze-dried hydrogels. The samples were ground with KBr and compressed into pellets prior to analysis. FTIR spectra were recorded using an FTIR spectrometer in the wavenumber range of 400–4000 cm^−1^.

### 4.4. Rheological Analysis

The rheological properties of the hydrogels were evaluated using an MCR 92 rotational rheometer (Anton Paar, Graz, Austria) equipped with a 40 mm diameter parallel-plate geometry at a gap of 1 mm, at 25 °C.

### 4.5. Morphological Analysis

The microstructure of the hydrogel cross-sections was examined using SEM (Hitachi SU8010 (Hitachi, Tokyo, Japan), Zeiss Gemini 300 (Zeiss, Oberkochen, Germany), and Hitachi S-4800 (Hitachi, Tokyo, Japan)). Briefly, the prepared hydrogel samples were rapidly frozen in liquid nitrogen and subsequently freeze-dried. The cross-sections of the hydrogels were then obtained by fracturing the samples under liquid nitrogen. To enhance electrical conductivity and ensure clear imaging, the fractured surfaces were sputter-coated with a thin layer of gold prior to SEM observation.

### 4.6. Swelling Ratio Tests

Freeze-dried hydrogels were immersed in PBS (pH 7.4) at 37 °C. At predetermined time points (0.5, 1, 2, 3 and 4 h), the samples were removed, excess surface moisture was blotted with filter paper, and the samples were weighed. The swelling ratio was calculated.

### 4.7. Particle Size, PDI and Zeta Potential Analysis

The average hydrated particle size, PDI and zeta potential of the hydrogel samples were measured by DLS using a nanoparticle size and zeta potential analyzer (Brookhaven Instruments Corporation, Nashua, NH, USA). Prior to measurement, the samples were diluted to a final concentration of 2 mg/mL. All measurements were performed in triplicate.

### 4.8. In Vitro Blood Compatibility Assay

The hemocompatibility of the hydrogels was evaluated by a hemolysis assay using mouse red blood cells (RBCs), following previously reported methods [34]. Briefly, whole blood was collected from mice and placed into anticoagulant tubes containing EDTA, and then washed five times with physiological saline by centrifugation to remove plasma and buffy coat. The isolated RBCs were then diluted with saline to obtain a 2% (*v*/*v*) erythrocyte suspension. Hydrogel–RBC mixtures were prepared by mixing the RBC suspension with hydrogels to achieve final hydrogel concentrations of 50%, 25%, 12.5%, 6.3%, 3.1%, and 0%. The mixtures were incubated at 37 °C for 1 h, during which the tubes were gently inverted twice to ensure sufficient contact between the hydrogels and RBCs. After incubation, the samples were centrifuged at 2000 rpm for 10 min. Physiological saline and 1% Triton X-100 were used as the negative and positive controls, respectively. The absorbance of the supernatant was measured, and the hemolysis rate was calculated using the following equation:

Hemolysis rate (100%) = (A_*n*_ − A_0_)/(A_1_ − A_0_) × 100% (1)

where A*_n_* represents the absorbance at the different concentrations of F127, F127−Lys−PCF127−Lys−PC, and F127−Lys−PCF127−Lys−PC@SGNPs; A_0_ is the absorbance of the negative control (saline); and A_1_ is the absorbance of the positive control (1% Triton X-100).

### 4.9. In Vitro Release of SGNPs from Composite Hydrogels in PBS

The *in vitro* release behavior of SGNPs from different hydrogel systems was evaluated using a dialysis method, with slight modifications based on the procedure reported by Liu et al. [35]. Briefly, free SGNPs, F127-SGNPs and F127−Lys−PCF127−Lys−PC@SGNPs were individually loaded into dialysis bags with a molecular weight cut-off of 35,000 Da. The dialysis bags were then immersed in 49 mL of PBS (pH 7.4) and incubated at 37 °C under gentle shaking to simulate physiological conditions. At predetermined time intervals, 500 μL of the release medium was withdrawn for analysis and immediately replaced with an equal volume of fresh PBS to maintain sink conditions. The amount of SGNPs released was quantitatively determined via high-performance liquid chromatography (HPLC) using an isocratic mobile phase consisting of 85% acetonitrile and 15% 0.1% phosphoric acid. The detection wavelength was set at 265 nm, with a column temperature of 25 °C and a flow rate of 1.0 mL/min. All experiments were performed in triplicate.

### 4.10. ABTS^+^ Radical Scavenging Assay

The ABTS radical scavenging activity of different hydrogel systems was evaluated using the ABTS assay, with slight modifications based on the method reported by Sathiyaseelan et al. [43]. Briefly, a 7.4 mM ABTS solution was mixed with 2.6 mM potassium persulfate (K_2_S_2_O_8_) and allowed to react in the dark at room temperature for 12 h to generate the ABTS radical cation (ABTS·^+^). The resulting ABTS·^+^ solution was then diluted with PBS to obtain an absorbance of 0.70 ± 0.02 at 734 nm. Subsequently, 3 mL of the ABTS·^+^ solution was incubated with F127, F127−Lys−PCF127−Lys−PC or F127−Lys−PCF127−Lys−PC@SGNPs hydrogels at different mass concentrations (0, 3.1, 6.2, 12.5, 25 and 50%) at 37 °C in the dark for 6 min. The absorbance of each sample was measured at 734 nm using a microplate reader. The ABTS·^+^ radical scavenging activity was calculated according to the following equation:

ABTS^+^ radical scavenging rate (%) = (A_0_ − A_*n*_)/A_0_ × 100 (2)

where A_0_ represents the absorbance of the blank control (ABTS^+^ solution) and A*_n_* represents the absorbance of the sample at 734 nm.

### 4.11. In Vitro Cell Experiments

A series of *in vitro* cell experiments were performed to systematically evaluate the cytocompatibility, protective effects against oxidative stress, and pro-migratory activity of the hydrogels. Human immortalized keratinocytes (HaCaT cells) were used as a representative skin cell model.

HaCaT cells were cultured in complete culture medium supplemented with 10% fetal bovine serum and 1% penicillin–streptomycin at 37 °C in a humidified atmosphere containing 5% CO_2_. Cells were seeded in 96-well plates at a density of 5 × 10^3^ cells per well and allowed to attach overnight before treatment.

To evaluate cytocompatibility, different hydrogel formulations were sterilized under UV irradiation and then incubated with HaCaT cells for 24 h. Cell viability was quantitatively assessed using a CCK-8 assay according to the manufacturer’s instructions. The absorbance was measured at 450 nm using a microplate reader, and cell viability was expressed as a percentage relative to the untreated control group. All CCK-8 assays were performed with *n* = 6 biological replicates for each experimental condition.

For the oxidative injury model, HaCaT cells were pre-incubated with different hydrogel formulations for 24 h. After removing the hydrogel-containing medium, the cells were exposed to 400 μM H_2_O_2_ for 4 h to induce oxidative stress. After the 4 h exposure period, the medium was removed, and fresh culture medium was added. Cell viability was then evaluated using the CCK-8 assay. Additionally, live/dead staining was performed using Calcein-AM/PI according to the manufacturer’s protocol, and fluorescence images were captured using a fluorescence microscope to qualitatively assess cell survival. These fluorescence staining experiments were performed with *n* = 3 biological replicates. ROS generation was also measured using the DCFH-DA assay, which detects intracellular ROS production.

To assess the effects of the hydrogels on cell migration, a scratch wound healing assay was conducted. HaCaT cells were seeded in 6-well plates and cultured until reaching approximately 90–100% confluence. A straight scratch was created using a sterile pipette tip, and the detached cells were gently removed by washing with PBS. Fresh medium containing different hydrogel formulations was then added. Images of the scratch area were captured at 0 h, 12h, 24 h, 36 h and 48 h.

### 4.12. In Vivo Experiments

All animal experiments were conducted in strict accordance with the Guidelines for the Care and Use of Laboratory Animals and were approved by the Animal Ethics Committee of Huaqiao University (Approval No. A2025092). Male C57BL/6 mice aged 6–8 weeks were housed under standard specific pathogen-free (SPF) conditions (22–25 °C, 12 h light/dark cycle) with free access to food and water. The mice were randomly assigned to control and treatment groups (*n* = 6). According to previously reported methods by 40. Wu et al. [41] with minor modifications, after anesthesia with 5% isoflurane, a full-thickness excisional wound with a diameter of approximately 1 cm was created on the dorsal skin of each mouse. Briefly, the wound was created using a sterile punch biopsy tool, and the wound edges were carefully trimmed to ensure a clean excision. The wounds were then treated daily with the corresponding hydrogel formulations according to the experimental groups. Specifically, F127−Lys−PCF127−Lys−PC@SGNPs hydrogel, which contains two concentrations of SGNPs (50 μM and 100 μM of sinomenine), was applied, and 200 μL of the hydrogel formulation was gently applied directly onto the wound site. After each treatment, the hydrogel was kept on a 37 °C warming plate to maintain its temperature and allow for gelation. The application of the hydrogel and photographing were performed at regular intervals each day. On days 4 and 9 post-surgery, wound tissues were harvested for histological evaluation using H&E staining and Masson’s trichrome staining.

### 4.13. Statistical Analysis

All data are presented as the mean ± standard deviation (SD, *n* = 3) unless otherwise stated. Statistical analyses were performed using GraphPad Prism 7.0 software (La Jolla, CA, USA). Comparisons among groups were conducted using one-way analysis of variance (ANOVA) followed by Tukey’s post hoc test. A value of *p* < 0.05 was considered statistically significant.

## Figures and Tables

**Table 1:** Chemical compositions and final concentrations of different hydrogel formulations.

	F127 (*w*/*v* %)	Lys (*w*/*v* %)	PC (*w*/*v* %)	SGNPs (Equiv. Sinomenine Concentration/mM)
F127−Lys−PCF127−Lys−PC	25	0.3/0.6	0.1/0.2/0.3	0
F127−Lys−PCF127−Lys−PC@SGNPs	25	0.3	0.1	1

Data are presented as mean ± SD (*n* = 3).

## Data Availability

The data presented in this study are available upon request from the corresponding author.

## Supplementary Materials

The following supporting information can be downloaded at https://www.mdpi.com/article/10.3390/gels12030191/s1, Figure S1. Characterization of SGNPs. (A) Physical mixture of sinomenine and gallic acid with visible undissolved solids. (B) Freshly prepared SGNPs showing a clear, homogeneous appearance without precipitation. (C) The physical mixture in water, exhibiting poor solubility. (D) SGNPs dispersed in water, demonstrating significantly enhanced water dispersibility. (E) TEM image of SGNPs, revealing a uniform spherical morphology and good dispersion; Figure S2. Solution appearance of PC at different concentrations. Flocculent precipitation was observed in systems with PC concentrations higher than 0.1%; Figure S3. Rheological properties of composite hydrogels with different lysine contents. (A) G′ as a function of strain for composite hydrogels containing 0.3% Lys, 0.3% Lys-loaded SGNPs, 0.6% Lys and 0.6% Lys-loaded SGNPs. (B) G″ as a function of strain. (C) tan δ of the hydrogels; Figure S4. Physicochemical characteristics of F127-based hydrated hydrogels. Particle size, zeta potential, and PDI of F127−Lys−PCF127−Lys−PC; Figure S5. Rheological characteristics of F127-based hydrogels. Crossover points of G′ and G″ for F127, F127−Lys−PCF127−Lys−PC and F127−Lys−PCF127−Lys−PC@SGNPs, indicating the sol–gel transition behavior; Figure S6. Quantitative porosity analysis of the hydrogels derived from SEM images using Image J (*n* = 3). Data are expressed as mean ± SD; Figure S7. Standard curve of sinomenine in the range of 1–5 mg/mL determined by HPLC; Table S1. Gelation time of F127 hydrogels at different polymer concentrations.

## Abbreviations

The following abbreviations are used in this manuscript:

DLS	Dynamic light scattering
F127	Pluronic F127
FTIR	Fourier transform infrared spectroscopy
G′	Storage modulus
G″	Loss modulus
HPLC	High-performance liquid chromatography
Lys	L-lysine
PC	Phosphatidylcholine
PDI	Polydispersity index
RBCs	Red blood cells
ROS	Reactive oxygen species
SEM	Scanning electron microscopy
SGNPs	Sinomenine–gallic acid nanoparticles
TGA	Thermogravimetric analysis
