# Supplementary material for: Injectable Thermosensitive Composite Hydrogels for Sustained Nanoparticle Delivery and Enhanced Wound Healing
Source: Gels. 2026 Feb 25;12(3):191. doi: 10.3390/gels12030191 (PMC13025013; doi:10.3390/gels12030191)
Supplement: Supplementary file 1 [file gels-12-00191-s001.zip › gels-4124513-supplementary.pdf]

Supporting Information for

Original article

Injectable Thermosensitive Composite Hydrogels for Sustained Nanoparticle Delivery and Enhanced Wound Healing

1. Supporting Figures S1–S7

2. Supporting Table S1

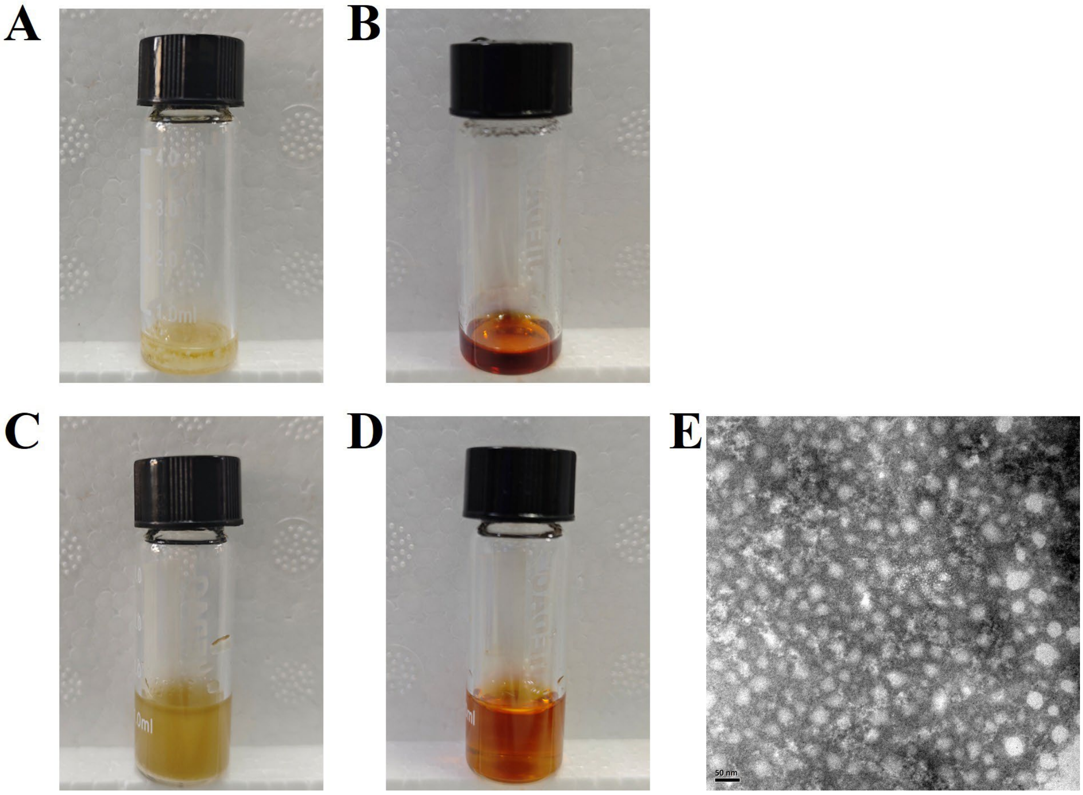

Figure S1. Characterization of SGNPs. (A) Physical mixture of sinomenine and gallic acid with visible undissolved solids. (B) Freshly prepared SGNPs showing a clear, homogeneous appearance without precipitation. (C) The physical mixture in water,

exhibiting poor solubility. (D) SGNPs dispersed in water, demonstrating significantly enhanced water dispersibility. (E) TEM image of SGNPs, revealing a uniform spherical morphology and good dispersion.

Methods: Preparation of SGNPs

SGNPs were synthesized via a streamlined one-step self-assembly strategy. Briefly, sinomenine and gallic acid (1:1 molar ratio) were dissolved in dimethyl sulfoxide (DMSO) and stirred vigorously at 60 °C for 4 h. This process facilitates the formation of stable supramolecular complexes through molecular self-assembly. To obtain the final aqueous dispersion, double-distilled water was added, ensuring the final DMSO concentration was maintained at a biocompatible level of 0.003% (v/v).

Results & Discussion: Characterization of SGNPs

The self-assembly process fundamentally alters the aqueous solubility and stability of the precursors. As shown in Figure S1C, the simple mixture of sinomenine and gallic acid (initially dissolved in DMSO) undergoes rapid re-precipitation upon dilution with water, leading to visible undissolved solids. In stark contrast, the self-assembled SGNPs exhibit excellent water dispersibility and remain as a transparent, stable dispersion without any precipitation (Figure S1D).

The morphological features were further characterized by TEM (Figure S1E), which reveals that SGNPs are uniform, spherical nanoparticles. This transformation from bulk crystalline phase to well-dispersed nanoparticles significantly enhances the processability of sinomenine within the hydrogel matrix. Given that the primary focus of this study is the hydrogel delivery system, the detailed characterization of SGNP formation is provided in the Supplementary Information.

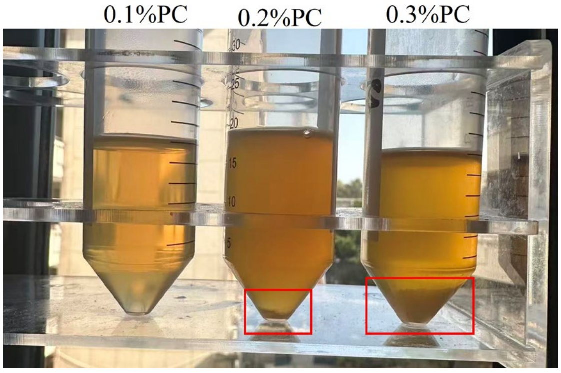

Figure S2. Solution appearance of PC at different concentrations. Flocculent precipitation was observed in systems with PC concentrations higher than 0.1%.

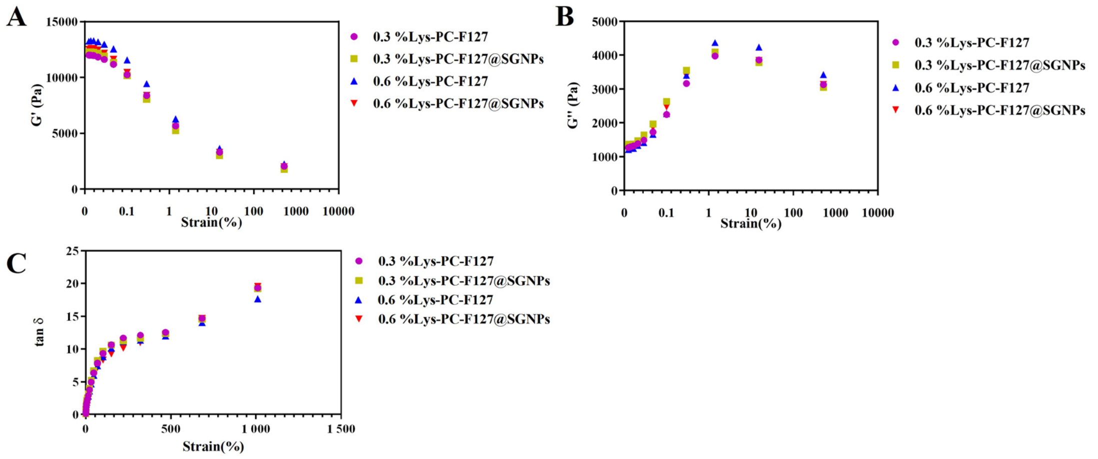

Figure S3. Rheological properties of composite hydrogels with different lysine contents. (A) G′ as a function of strain for composite hydrogels containing 0.3% Lys, 0.3% Lys-loaded SGNPs, 0.6% Lys and 0.6% Lys-loaded SGNPs. (B) G″ as a function of strain. (C) tan δ of the hydrogels.

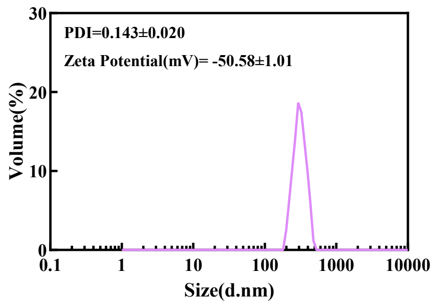

Figure S4. Physicochemical characteristics of F127-based hydrated hydrogels. Particle size, zeta potential, and PDI of F127-Lys-PC.

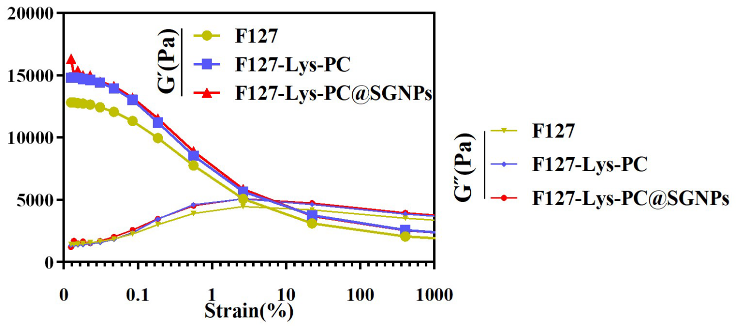

Figure S5. Rheological characteristics of F127-based hydrogels. Crossover points of G′ and G″ for F127, F127-Lys-PC and F127-Lys-PC@SGNPs, indicating the sol–gel transition behavior.

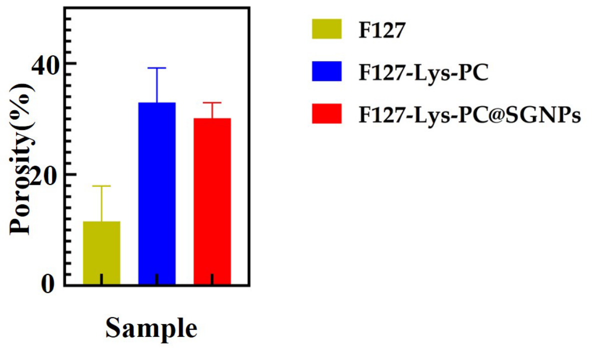

Figure S6. Quantitative porosity analysis of the hydrogels derived from SEM images using Image J (n=3). Data are expressed as mean ± SD.

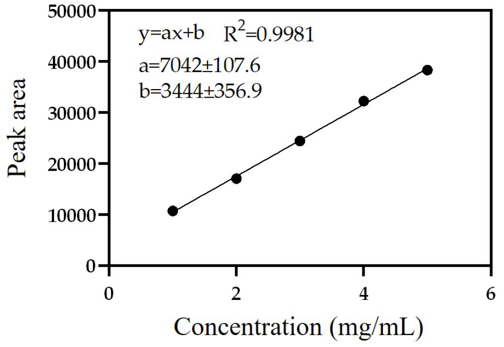

Figure S7. Standard curve of sinomenine in the range of 1–5 mg/mL determined by HPLC.

Table S1. Gelation time of F127 hydrogels at different polymer concentrations.

F127 content (%)	15	20	25	30
Gelation time (s)	931±61.2	461.7±25.2	234.3±26.1	136.7±25.4

Data are presented as mean ± SD (n = 3).
